# Dietary n-3 Polyunsaturated Fatty Acids (PUFA) Decrease Obesity-Associated Th17 Cell-Mediated Inflammation during Colitis

**DOI:** 10.1371/journal.pone.0049739

**Published:** 2012-11-16

**Authors:** Jennifer M. Monk, Tim Y. Hou, Harmony F. Turk, Brad Weeks, Chaodong Wu, David N. McMurray, Robert S. Chapkin

**Affiliations:** 1 Program in Integrative Nutrition & Complex Diseases, Texas A&M University, College Station, Texas, United States of America; 2 Department of Nutrition & Food Science, Texas A&M University, College Station, Texas, United States of America; 3 Department of Biochemistry and Biophysics, Texas A&M University, College Station, Texas, United States of America; 4 Department of Veterinary Pathobiology, Texas A&M University, College Station, Texas, United States of America; 5 Vegetable & Fruit Improvement Center, Texas A&M AgriLife, College Station, Texas, United States of America; 6 Department of Microbial and Molecular Pathogenesis, Texas A&M University System Health Science Center, College Station, Texas, United States of America; Virginia Tech, United States of America

## Abstract

Clinical and experimental evidence suggests that obesity-associated inflammation increases disease activity during colitis, attributed in part to the effects of Th17 cells. Using a model of concurrent obesity and colitis, we monitored changes in critical immune cell subsets and inflammatory biomarker expression in three key tissues: visceral adipose tissue, colon (local inflammatory site) and spleen (systemic inflammatory site), and we hypothesized that n-3 PUFA would reduce the percentage of inflammatory immune cell subsets and suppress inflammatory gene expression, thereby improving the disease phenotype. Obesity was induced in C57BL/6 mice by feeding a high fat (HF) diet (59.2% kcal) alone or an isocaloric HF diet supplemented with fish oil (HF-FO) for 12 weeks. Colitis was induced via a 2.5% trinitrobenzene sulfonic acid (TNBS) enema. The HF-FO diet improved the obese phenotype by reducing i) serum hormone concentrations (leptin and resistin), ii) adipose tissue mRNA expression of inflammatory cytokines (MCP-1, IFNγ, IL-6, IL17F and IL-21) and iii) total (F4/80^+^ CD11b^+^) and inflammatory adipose tissue M1 (F4/80^+^ CD11c^+^) macrophage content compared to HF (*P*<0.05). In addition, the HF-FO diet reduced both colitis-associated disease severity and colonic mRNA expression of the Th17 cell master transcription factor (RORγτ) and critical cytokines (IL-6, IL-17A, IL-17F, IL-21, IL-23 and IFNγ) versus HF (*P*<0.05). Compared to HF, the percentage of both splenic Th17 and Th1 cells were reduced by the HF-FO group (*P*<0.05). Under *ex vivo* polarizing conditions, the percentage of HF-FO derived CD4^+^ T cells that reached Th17 cell effector status was suppressed (*P* = 0.05). Collectively, these results indicate that n-3 PUFA suppress Th1/Th17 cells and inflammatory macrophage subsets and reconfigure the inflammatory gene expression profile in diverse tissue sites in obese mice following the induction of colitis.

## Introduction

In the United States the prevalence of obesity is reported to be approximately 35% among adults [1]. Obesity is associated with low-grade chronic inflammation occurring within the adipose tissue and changes in immune cell populations give rise to altered inflammatory adipokine and cytokine profiles which, in part, induce skeletal muscle and hepatic insulin resistance, thereby causally linking obesity and type-2 diabetes [2], [3]. Interestingly, fish oil (FO) derived long chain n-3 polyunsaturated fatty acids (PUFA), specifically eicosapentaenoic acid (EPA) and docosahexaenoic acid (DHA), have been shown to favorably impact obesity-associated pathologies, including adipose tissue inflammation, insulin resistance, lipid metabolism and hepatic steatosis [4], [5], [6], [7], [8], [9], [10], [11], [12]. Additionally, supplementation of n-3 PUFA in models of high fat diet-induced obesity results in reduced insulin [4], [6] and leptin [6] levels, and increased adiponectin levels [5], [6], [12].

It is estimated that 50% of inflammatory bowel disease (IBD) subjects utilize self-prescribed complementary alternative medicines/diets, such as FO [13], whose anti-inflammatory effects have been shown to enhance remission of chronic intestinal inflammation [14]. In IBD, activation of two inflammatory mucosal CD4^+^ T cell subsets, Th1 and Th17 cells, plays a central role in the induction and persistence of chronic inflammation [15], [16], [17], [18], [19], [20], and antagonism of the Th17 cell pathway is associated with reduced disease severity [21], [22]. n-3 PUFA have been shown to antagonize the Th1 subset [23], [24] and reduce disease severity in dextran sodium sulfate (DSS)-induced colitis, while concomitantly suppressing systemic (splenic) and local (colon lamina propria) Th17 cell accumulation, in part, by decreasing mucosal expression of critical Th17 cell-related cytokines [25].

In obesity, mesenteric adipose tissue-derived inflammatory mediators contribute to ongoing intestinal inflammation [26], [27]. In Crohn’s disease (CD), mesenteric obesity is implicated in the disease pathogenesis, wherein fat wrapping and mesenteric adipose tissue hyperplasia are long established and consistent features in surgical specimens [27], [28], [29], [30]. There is significant intra-abdominal fat accumulation (i.e., creeping fat) in CD patients [31], and histological analysis has revealed abnormalities in the mesenteric adipose tissue, including pronounced immune cell infiltration, fibrosis, perivascular inflammation and vessel thickening [28], [29], [30]. Ultimately, the hypertrophied mesenteric adipose that surrounds the intestine [29] contributes actively to disease severity by increasing the inflammatory milieu comprised of cytokines (i.e., IL-6), chemokines (MCP-1) and immunomodulatory hormones (i.e., leptin and resistin) which may sustain or perpetuate inflammatory responses in CD patients [26], [32], [33]. Moreover, the increased visceral adipose mass in obese CD patients may predispose them to a more aggressive clinical course [31], [34]. For example, disease severity was increased in obese CD patients, making them more prone to develop active disease (e.g., anorectal complications such as anoperineal abscesses or fistulas) and require hospitalization as compared to non-obese CD patients [27], [31], [35]. In animal models, co-morbid diet-induced obesity and trinitrobenzene sulfonic acid (TNBS)-induced colitis has been associated with activation of the inflammatory Th17 cell subset, resulting in more severe colonic histological damage and increased in vitro production of IL-17 [36]. Since n-3 PUFA antagonize Th17 cells during DSS-induced colitis [25], we hypothesized that in a combinatory model of diet-induced obesity and TNBS-induced colitis, dietary FO would have a beneficial impact on the overall disease phenotype, in part, by suppressing (local) colonic Th17 cell polarizing cytokines and by reducing inflammatory T cell subsets systemically. Therefore, in this study, we monitored changes in critical immune cell subsets and inflammatory biomarker (cytokine/adipokine) expression in three key tissues: visceral adipose tissue, which increases in mass during obesity and surrounds the inflamed gastrointestinal tract [26], [37], the colon (local site of colitis-induced inflammation) and the spleen (systemic site of inflammation). Overall, supplementation of n-3 PUFA during concurrent obesity and TNBS-induced colitis improved disease outcomes and suppressed both inflammatory immune cell populations locally and systemically while concomitantly reconfiguring the inflammatory gene expression profile in multiple tissue sites.

## Materials and Methods

### Ethics Statement

All procedures adhered to U.S. Public Health Service Policy and were approved by the Institutional Animal Care and Use Committee at Texas A&M University.

### Animals and Diet

Specific pathogen-free male C57BL/6 mice were maintained under barrier conditions for 12 weeks and fed one of two isocaloric high fat diets (59.2% kcal) which contained an equal amount of lard (39% kcal) but differing levels of n-3 and n-6 PUFA from vacuum-deodorized menhaden fish oil (FO) and corn oil (CO), respectively [high fat (HF): 20.2% kcal corn oil (CO) or high fat+fish oil (HF-FO): 14.2% kcal FO+6% kcal CO]. Control mice consumed a low fat (LF) diet (10.4% kcal) [LF: 4.6% kcal lard+5.8% kcal CO]. The content of bioactive n-3 PUFA in the HF-FO supplemented diet was 3.6% kcal as DHA and EPA combined, which is consistent with the level consumed by the Greenland Inuit [38]. The diets were modeled after the commercially available high fat and low fat formulations that have been used elsewhere to produce diet-induced obesity [39], [40]. The diet composition is described in **Table S1**. Diets were replaced daily to prevent oxidation.

### Colitis Induction and Histological Scoring

Colon inflammation was induced by exposure to 2,4,6-trinitro benzene sulfonic acid (TNBS; Sigma Aldrich) as described previously [41]. In brief, mice were individually housed and 100 µl of a 1% (w/v) pre-sensitizing dosage of TNBS in the vehicle [4∶1 volume ratio of acetone and olive oil (Azienda)] was applied topically onto a shaved 1.5 × 1.5 cm field between the shoulders. The site was selected to prevent the animals from ingesting the TNBS which could induce oral tolerance [41]. After 7 d, mice were anesthetized with isoflurane to effect and were under anesthesia for ≤5 min during which time a 100 µl enema containing 2.5% (w/v) TNBS in a 1∶1 volume ratio of water and absolute ethanol was administered. To minimize excretion of the TNBS solution, animals were inverted for 1 min following completion of the enema and placed head down at a 60° incline for approximately 5 min. Vehicle control mice were exposed to the presensitization vehicle topically, followed by an enema consisting of 1∶1 volume ratio of water and absolute ethanol. All mice were sacrificed 3 d post-enema. Body weights and food intakes were monitored throughout the study. Mice were sacrificed by CO_2_ asphyxiation and colons were excised (distal to the cecum and proximal to the anus), flushed with sterile PBS and the mucosa was scraped from one longitudinal half. The other half of the colon was fixed in 4% paraformaldehyde, swiss-rolled, paraffin embedded and stained with hematoxylin and eosin. The degree of colon inflammation (score 0–3) and injury (score 0–3) were graded and combined for a total score in a blinded manner by a board-certified pathologist (B. Weeks) in accordance with the criteria outlined previously [25], [42].

### Serum Hormone Profiles

Orbital plexus blood was collected prior to sacrifice, allowed to clot at room temperature for 30–45 min, then centrifuged at 14000×g for 4 min and the resulting serum was stored at −80°C. Serum levels of insulin, leptin, resistin and adiponectin were measured using the Milliplex MAP mouse metabolic hormone magnetic bead panel (EMD Millipore) and samples were run on the Bio-Plex 200 System using Bio-Plex Manager 6.0 software (BioRad).

### Isolation of Adipose Tissue and Stromal Vascular Cells

Adipose tissue was isolated and weighed from three individual fat pads (mesenteric, epididymal and perinephric), which combined represent total visceral adipose. Stromal vascular cells (SVC) were isolated by collagenase digestion as described previously [43]. Antibodies used in the subsequent SVC flow cytometry analysis were APC-F4/80 (clone: Bm8, eBioscience), PE-CD11b and APC-CD11b (clone: M1/70, eBioscience), PE-CD11c (clone: HL3, BD Biosciences), APC-CD206 (clone: C068C2, BioLegend). M1 and M2 macrophages were defined as F4/80^+^ CD11c^+^ and F4/80^+^ CD206^+^, respectively [44], [45].

### Flow Cytometry Analysis of Treg, Th1 and Th17 Cells

Splenic mononuclear cells were isolated as described [46] and surface and intracellular staining performed as reported previously [25]. Cells were preincubated with a FcγR blocking monoclonal mAb (1 µg/mL) (2.4G2, BD Pharmingen) for 10 min on ice, followed by surface (CD4) and intracellular (FOXP3, IL-17A or IFNγ) staining. Antibodies used were APC-anti-CD4 (L3T4), PE-anti-FOXP3 (FJK-16s) (eBioscience), PE-anti-IL-17A (TC11-18H10) and PE-anti-IFNγ (XMG1.2) (BD Pharmingen). Flow cytometric analysis was conducted using an Accuri C6 flow cytometer (Accuri Cytometers).

### Splenic T Cell *in vitro* Polarization Conditions

Spleens were removed aseptically and CD4^+^ T cells were isolated by positive selection using magnetic CD4 (L3T4) microbeads according to the manufacturer’s instructions (Miltenyi Biotec). Cell purity exceeded 90% as described previously [47]. 2×10^5^ viable CD4^+^ T cells (assessed via trypan blue exclusion) were added to each well of a round bottom 96-well plate (BD Bioscience) in a final volume of 200 µl of complete RPMI [RPMI 1640 medium with 25 mmol/L HEPES (Irvine Scientific), 50 µM 2-mercaptoethanol (Sigma Aldrich), 5% fetal bovine serum (Irvine Scientific), 2 mM GlutaMAX (Gibco), penicillin 100 U/mL and streptomycin 0.1 mg/mL (Gibco), henceforth “complete medium”]. All cultures were stimulated with 5 µg/ml of plate-bound anti-CD3 (145-2C11, BD Bioscience) plus 5 µg/ml of soluble anti-CD28 (37.51, eBioscience). For Treg polarizing conditions, cultures were supplemented with 2 ng/ml TGF- β1 (BioLegend). For Th17 cell polarizing conditions, cultures were supplemented with 2 ng/ml TGF-β1, 10 ng/ml IL-6, 20 ng/ml IL-23 (BioLegend), 10 µg/ml anti-IFNγ (AN-18, eBioscience) and 10 µg/ml anti-IL-4 (11B11, eBioscience). Cells were incubated at 37°C for 72 h and subsequently stimulated with 1X brefeldin A (diluted from a 10X stock, eBioscience), 1 µM ionomycin (EMD Chemicals) and 50 ng/ml PMA (Sigma Aldrich) for an additional 5 hours prior to intracellular staining with PE-anti-FOXP3 (FJK-16s) (eBioscience) or PE-anti-IL-17A (TC11-18H10) (BD Pharmingen) antibodies.

### RNA Isolation and Measurement of mRNA Expression

RNA was isolated from vehicle control and TNBS-treated mice using the RNA 4-PCR kit (Ambion) for colon mucosa scrapings and the ToTALLY RNA kit (Ambion) for adipose tissue. Real-time RT-PCR was used to quantify mRNA expression and amplification was performed using the Taqman Universal PCR master mix (Applied Biosystems). Taqman gene expression kits (Applied Biosystems) were used for amplification, namely IL-1β (Mm00434228_m1), IL-6 (Mm00446190_m1), IL-17A (Mm00439618_m1), IL-17F (Mm00521423_m1), IL-21 (Mm00517640_m1), IL-23 (Mm00518984_m1), IFNγ (Mm01168134_m1), IL-27 (Mm00461162_m1), TNFα (Mm00443260_g1), CCL2 (MCP-1, Mm00441242_m1), IL-10 (Mm00439614_m1), TGFβ1 (Mm01178820_m1), Rorc (RORγτ, Mm01261022_m1), Tbx21 (T-bet, Mm00450960_m1), FOXP3 (Mm00475162_m1). Amplification of mRNA (fluorescence) was recorded over 40 cycles and the corresponding cycle numbers (Ct) were used to calculate mRNA expression according to the calculation: 2^(40−Ct)^. Target gene expression was normalized to ribosomal 18S expression (Mm03928990_g1).

### Statistics

The predetermined upper limit of probability for statistical significance throughout this investigation was *P*<0.05, and analyses were conducted using the SAS system (SAS Institute) for Windows (version 9.0). Data were subjected to two-way ANOVA (main effects: diet and treatment) followed, if justified, by testing using Least Squares Means. Data sets not exhibiting a normal distribution were subjected to the Kruskal-Wallis test (χ^2^ approximation) followed, if justified, by the statistical probability outcome (*P*<0.05) using Wilcoxon two-sample testing.

## Results

### HF Diet Induces an Obese Phenotype which is Ameliorated by n-3 PUFA Supplementation

**Figure 1:**
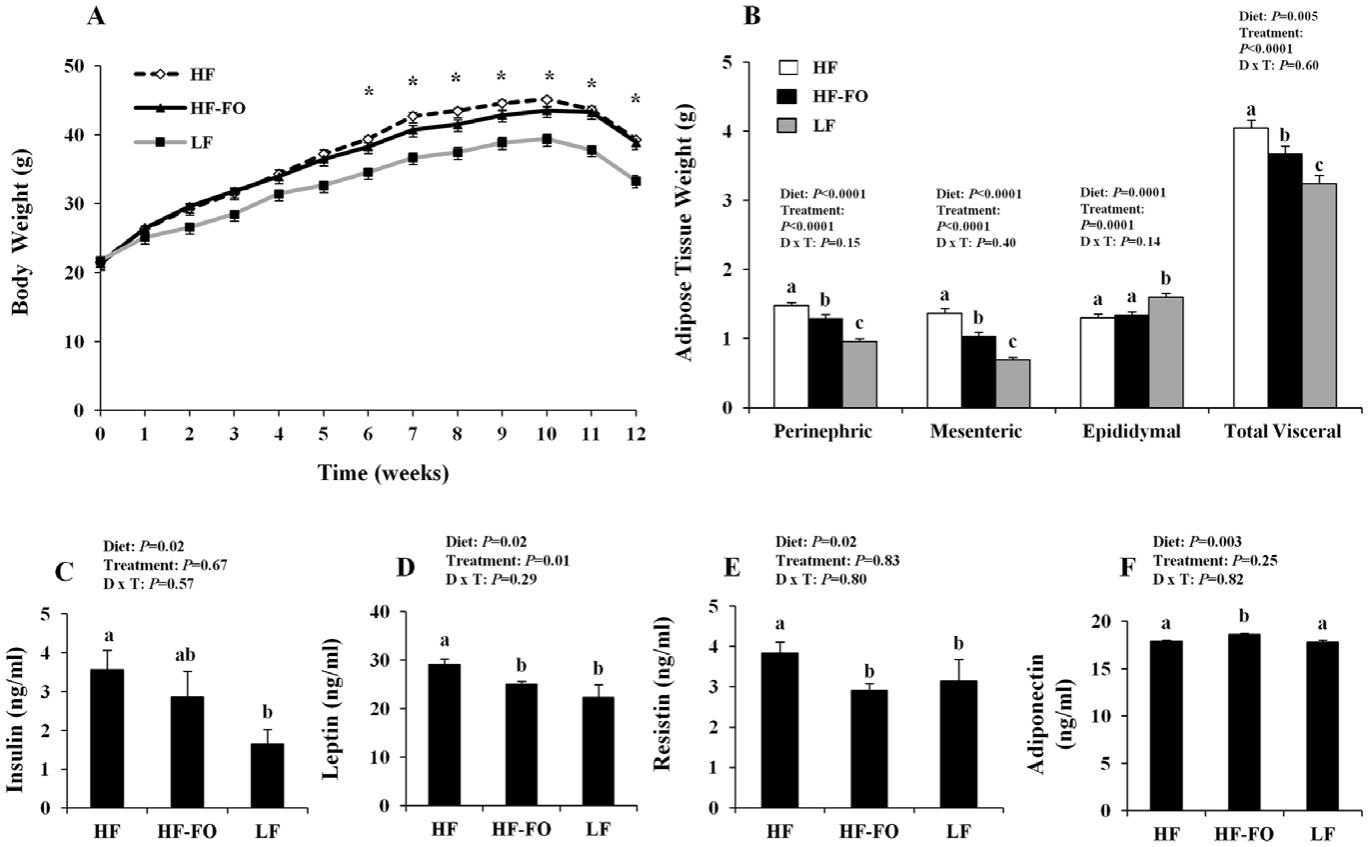
Characterization of the diet-induced obese phenotype. C57BL/6 mice were fed a high diet (HF), high fat diet supplemented with FO (HF-FO) or a low fat (LF) control diet for 12 weeks (n = 12−17 TNBS-treated and 4–6 vehicle controls/diet). Mice were presensitized with 1% TNBS or vehicle control (week 11), followed by a 2.5% TNBS enema or vehicle control (week 12) and sacrificed 3 d post-TNBS. A) Changes in body weight over time. B) Visceral adipose tissue weight from individual visceral depots (perinephric, mesenteric and epididymal) or combined (total visceral adipose). Serum concentrations of C) insulin, D) leptin, E) resistin and F) adiponectin. All data were analyzed by two-way ANOVA (main effects: diet and treatment) and *P*-values are shown. Bars represent means ± SEM and statistical significance was (*P*≤0.05). Panel A) asterisk (*) indicates statistically significant time points where the HF and HF-FO groups differed from LF (*P*≤0.05). Panels B-F) bars not sharing a common letter differ (*P*≤0.05).

Body weights were monitored throughout the study and did not differ between mice consuming the HF and HF-FO diets (*P*>0.05); however, during weeks 6–12 of dietary intervention, both HF and HF-FO-fed mice gained more weight compared to LF-fed animals (*P*<0.05) (**Figure 1A**). Food intake did not differ between the HF and HF-FO groups at any point throughout the duration of the study (*P*>0.05). During the TNBS pre-sensitization period (week 11) and following TNBS enemas (week 12), all dietary groups experienced a modest degree of weight loss that is typically associated with TNBS treatment [48]. Analysis of visceral adipose tissue weights (**Figure 1B**) revealed that compared to HF-FO mice, the HF diet group exhibited higher perinephric and mesenteric adipose tissue weights. As expected, adipose tissue weights in both high fat diets (HF and HF-FO) were elevated relative to LF (*P*<0.05). Conversely, there was no difference in epididymal adipose weights between HF and HF-FO-fed mice (*P* = 0.25). Interestingly, LF-fed animals had a modestly higher epididymal fat content (*P*<0.05); however, elevated or unchanged epididymal fat weights have been reported previously in low fat diet fed rodents [9], [11]. Overall, combined visceral adipose tissue mass (mesenteric, perinephric and epididymal) was increased in both the HF and HF-FO groups relative to the LF group (*P*<0.05), and the HF-FO group exhibited a lower total visceral adipose tissue mass compared to the HF diet. Biochemical evidence of an obese phenotype was assessed by monitoring changes in systemic hormone concentrations (**Figure 1C–F**). Compared to LF, the HF group exhibited higher insulin, leptin and resistin (*P*<0.05) concentrations whereas adiponectin levels did not differ (*P*>0.05). These outcomes are consistent with previous reports in both rodent HF-diet induced obesity [5], [6], [12] and in obese humans [49], [50]. Interestingly, the HF-FO group exhibited an intermediate hormonal phenotype, in which circulating levels of leptin and resistin were significantly reduced relative to HF (*P*<0.05) and did not differ from levels observed in the LF group (*P*>0.05). HF-FO insulin levels did not differ from those in the HF or LF groups (*P*>0.05). Interestingly, serum adiponectin, an anti-inflammatory hormone typically reduced during obesity, exhibited a modest but significantly higher concentration in the HF-FO group relative to both the HF and LF diets (*P*>0.05), which is consistent with previous reports with n-3 PUFA supplementation [5], [6], [12]. Collectively, these data confirm the ability of the semi-purified HF diet to recapitulate critical aspects of the obese phenotype relative to LF. In addition, n-3 PUFA supplementation to the HF diet mitigated the severity of the obesity induced changes in the circulating hormone profile and the accumulation of visceral adipose tissue.

### n-3 PUFA Reduce Adipose Tissue Macrophage Infiltration by Decreasing Both the M1 and M2 Subsets

**Figure 2:**
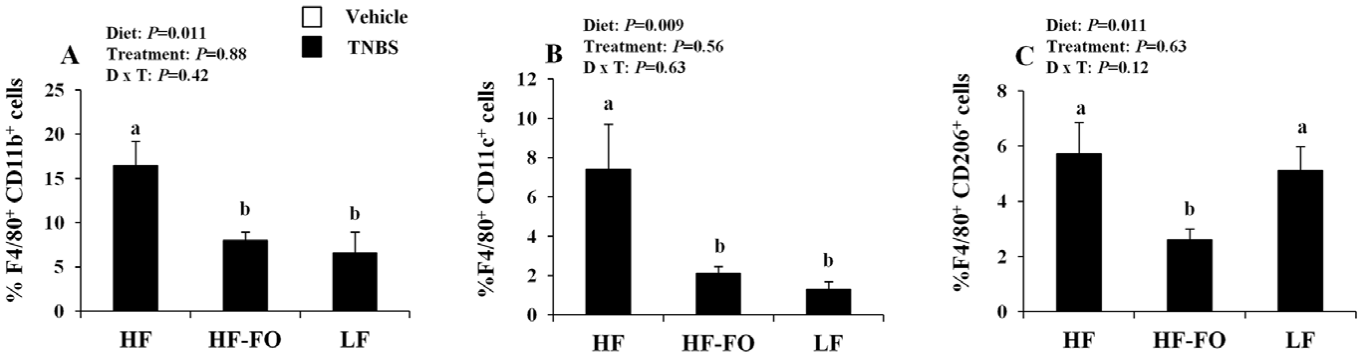
Visceral adipose tissue macrophage infiltration. Stromal vascular cells (SVC) were isolated and quantified from total visceral adipose tissue (HF and HF-FO groups, n = 3 vehicle controls and 6–8 TNBS-treated mice, LF n = 4 pooled samples comprised of 3–4 mice/treatment). A) percentage of F4/80^+^ CD11b^+^ cells (total macrophages), B) percentage of F4/80^+^ CD11c^+^ cells (M1 macrophages), C) percentage of F4/80^+^ CD206^+^ cells (M2 macrophages). Data were analyzed by two-way ANOVA (main effects: diet and treatment) and bars represent mean values ± SEM. Bars not sharing a common letter are significantly different (*P*≤0.05).

A hallmark of obesity is the increased infiltration of macrophages into the inflamed adipose tissue [51], [52], [53]. The SVC population is comprised of multiple cell types including preadipocytes, mesenchymal stem cells, endothelial precursor cells and immune cells [54], therefore, we initially characterized the total adipose tissue macrophage population [52]. Representative dot plots of total, M1 and M2 macrophage subsets are presented in **Figure S1**. Consistent with previous reports [51], [52], [53], there was an obesity-associated increase in adipose tissue macrophage content, i.e., the percentage of F4/80^+^ CD11b^+^ cells, which was increased in the HF group relative to LF (*P*<0.05, **Figure 2A**). Interestingly, compared to HF, the HF-FO diet prevented the obesity-associated increase in adipose macrophage content, reducing the percentage of total adipose F4/80^+^ CD11b^+^ macrophages by 52% (*P*<0.05). Further analysis of specific macrophage subsets (classically activated, M1, and alternatively activated, M2 subsets, **Figures 2B & C**, respectively) revealed that obese mice (HF diet) exhibited a higher percentage of inflammatory M1 macrophages (F4/80^+^ CD11c^+^) compared to the LF diet (*P*<0.05). In contrast, the obesity-associated perturbation was prevented by n-3 PUFA supplementation which reduced the percentage of adipose M1 macrophages compared to HF by 72% but did not differ from the LF group (*P*>0.05). Interestingly, there was no obesity-associated increase in the percentage of M2 macrophages (F4/80^+^ CD206^+^), as the HF and LF diets did not differ (*P*>0.05). In the HF-FO group, however, the percentage of M2 macrophages was also significantly reduced by 55% in comparison to the HF group. Total macrophage content in the adipose tissue, therefore, was reduced by n-3 PUFA without a preference for a particular macrophage subset.

### n-3 PUFA Suppress the Obesity-associated Adipose Tissue Inflammatory Gene Expression Profile

Within the adipose tissue, changes in the local inflammatory cytokine milieu were assessed at the mRNA level. For all genes examined, colitis status had no independent effect on adipose tissue gene expression in any dietary group (i.e., vehicle control versus TNBS, *P*>0.05); therefore, the effect of diet alone on adipose tissue gene expression is presented in **Table 1**. Consistent with the ability of n-3 PUFA to reduce the percentage of adipose tissue infiltrating macrophages during obesity (**Figure 2**), adipose mRNA expression of MCP-1, a macrophage chemotactic signal, was reduced by 44% in the HF-FO group relative to HF alone (*P* = 0.001). Additionally, compared to HF, n-3 PUFA supplementation reduced the expression of a key inflammatory cytokine, IL-6 (*P* = 0.008) by 29%. However, gene expression of TNFα and IL-1β did not differ between dietary groups (*P*>0.05). Expression of the classic inflammatory Th1 cytokine, IFNγ was down regulated by 83% in the HF-FO group compared to HF (*P* = 0.02). Consistent with this finding, expression of the master transcription factor associated with Th1 cells, Tbet, was also reduced by n-3 PUFA (*P* = 0.03). Cytokines related to Th17 polarization and maintenance were similarly affected, with mRNA levels of IL-17F and IL-21 reduced by 65% (*P* = 0.004) and 57% (*P* = 0.03), respectively, in the HF-FO group compared to HF alone. IL-17A mRNA was undetectable, and there was no difference between dietary groups in adipose mRNA expression of IL-23 (*P* = 0.42) and IL-27 (*P* = 0.76). Expression of the transcription factor that drives the polarization of Th17 cells, RORγτ, exhibited a non-significant trend towards suppression by n-3 PUFA during obesity (HF-FO versus HF, *P* = 0.08). Adipose expression of the Treg specific transcription factor Foxp3 was unaffected by diet (*P* = 0.81). Consistent with reduced inflammatory potential, adipose mRNA expression of the anti-inflammatory cytokine IL-10 was upregulated in the HF-FO group relative to HF by 42% (*P* = 0.05) whereas expression of TGF-β1 mRNA was unaffected by diet (*P* = 0.93). Collectively, these data demonstrate that n-3 PUFA suppress the transcription of inflammatory cytokines which drive and sustain the local inflammatory microenvironment within adipose tissue.

**Table 1:** Visceral adipose tissue mRNA expression^1^.

Gene	HF	HF-FO	LF	Diet: *P*-value
MCP-1	5.01±0.49^a^	2.82±0.3^b^	1.91±0.61^b^	0.001
TNFα	11.00±1.28	7.81±0.63	8.86±4.01	0.10
IFNγ	11.97±4.23^a^	2.09±0.74^b^	4.81±2.78^ab^	0.02
IL-1β	2.02±0.51	2.22±0.46	1.39±0.38	0.91
IL-6	1.33±0.41^a^	0.94±0.33^b^	2.29±0.38^b^	0.008
IL-10	4.17±0.35^a^	7.14±1.55^b^	5.34±3.03^a^	0.05
IL-17F	1.03±0.32^a^	0.36±0.15^b^	1.72±1.21^ab^	0.004
IL-21	11.42±2.95^a^	4.95±1.31^b^	0.95±0.12^b^	0.03
IL-23	10.13±1.48	7.60±1.3	9.75±1.51	0.42
IL-27	8.35±1.02	9.53±1.15	9.09±2.47	0.76
TGF-β1	8.81±0.85	8.29±0.93	8.62±1.73	0.93
RORγτ	9.08±3.21	3.75±0.54	3.29±0.94	0.08
T-bet	6.58±1.06^a^	3.93±0.53^b^	3.92±0.50^b^	0.03
Foxp3	1.62±0.41	1.06±0.22	1.26±0.54	0.81

1 Values are means ± SEM (n = 5−10/dietary group). Data were analyzed by two-way ANOVA (main effects: diet and treatment). For all genes, there was no effect of treatment (i.e., TNBS versus vehicle, *P*>0.05), therefore, only the main effect of diet is shown. Within individual genes, values not sharing a lower case letter denote significant differences (*P*≤0.05). Data were normalized to ribosomal 18S.

### HF-FO-fed Mice are more Resistant to TNBS-induced Colonic Inflammation

**Figure 3:**
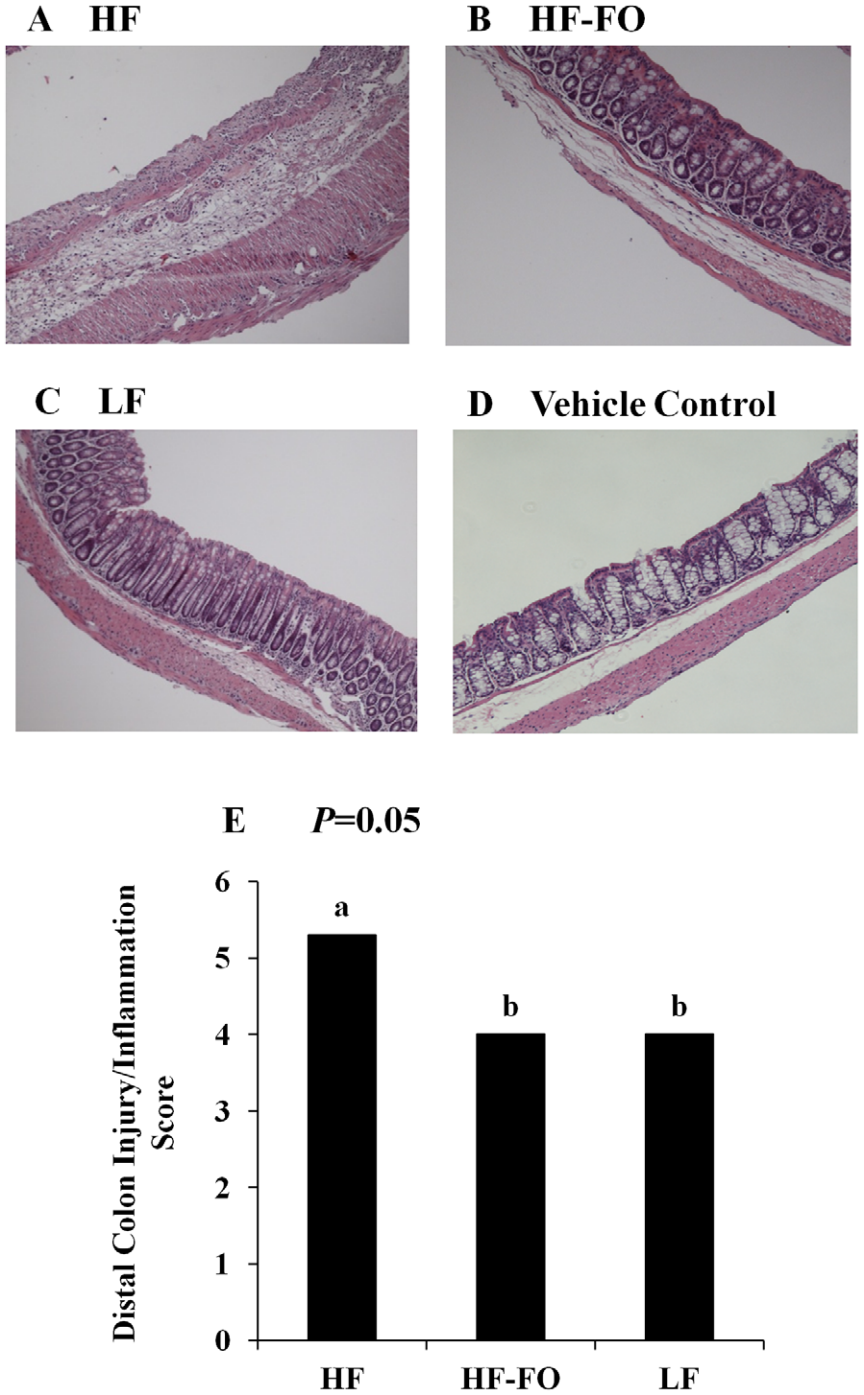
Colon histological disease scores for TNBS-treated mice. Colonic mucosal injury (0–3) and inflammation (0–3) scores were assessed in a blinded manner by a board-certified pathologist (B. Weeks) and combined for a total score (0–6). Representative images (100 × magnification) are shown for the HF, HF-FO and LF TNBS-treated groups, respectively (panels A-C) and a representative image of a HF vehicle control (panel D) is shown. E) Combined injury/inflammation histological score within the distal colon (n = 10−14 TNBS treated mice/diet). Data were analyzed using the Kruskal-Wallis test followed by Wilcoxon two-sample testing, and bars represent median values. Bars not sharing a common letter are significantly different (P≤0.05).

The degree of colon inflammation and injury following exposure to TNBS was assessed based on gross changes observed within the colon histological architecture in a blinded manner by a board-certified pathologist (B. Weeks). There was no effect of diet on vehicle control treated colon histological scores (*P* = 0.11), and as expected, TNBS treatment increased both colon inflammation and injury scores, independently of diet (*P*>0.05) relative to vehicle controls (data not shown). Representative colon images from TNBS-treated mice from each dietary group (**Figure 3A–C**) and from a representative vehicle control-treated mouse (**Figure 3D**) are shown. Within the distal colon of TNBS-treated mice, there was a significant effect of diet (*P* = 0.05) on the histological disease score, which was significantly elevated in the HF group relative to LF (**Figure 3E**). This outcome is consistent with previous findings demonstrating that obesity is associated with a more severe response to TNBS-induced colitis [36]. Interestingly, the HF-FO group exhibited a lower histological disease score compared to the HF group (*P* = 0.05) but did not differ from the LF group (*P* = 0.85), indicating that n-3 PUFA supplementation prevented the obesity-associated enhanced inflammatory response to TNBS-induced colitis. These outcomes are consistent with the ability of dietary n-3 PUFA to enhance the resolution of inflammatory processes in other colitis models [25], [42].

### n-3 PUFA Modulate the Colonic Mucosal Cytokine Microenvironment in a Manner Consistent with Reduced Th17 Cell Activation

To gain insight into how effector T cell populations are changing in the context of concurrent obesity and colitis, the expression of critical transcription factors associated with specific T cell subsets in the colonic scraped mucosa, i.e., FOXP3 (Tregs), Tbet (Th1 cells) and RORγτ (Th17 cells) and critical cytokines that influence T cell polarization and function were assessed. For each gene, colonic mucosal basal (i.e., vehicle control) mRNA expression did not differ between dietary groups (*P*>0.05, **Table S2**). As expected, exposure to TNBS upregulated mucosal gene expression relative to vehicle controls (*P*<0.05) in all dietary groups; therefore, the outcomes between dietary groups among TNBS-treated mice only are shown in **Table 2**. During TNBS-induced colitis, there was an obesity-associated 43% increase in mucosal expression of RORγτ mRNA (LF versus HF, *P* = 0.03). Interestingly, supplementation of n-3 PUFA blocked the obesity-associated increase in RORγτ expression, which was significantly reduced compared to the HF group but did not differ from LF. These data demonstrate that expression of RORγτ, a master transcription factor that directs both the differentiation of Th17 cells and the expression of hallmark Th17 cytokines [55], was modulated by n-3 PUFA in a manner consistent with a reduced Th17 cell response in the colon. Tbet, the master transcription factor driving Th1 cell polarization, similarly exhibited an obesity-associated elevation in mucosal mRNA expression during TNBS-induced colitis (HF vs. LF, *P* = 0.02). However, Tbet mRNA expression in the HF-FO group did not differ from either HF or LF (*P*>0.05), but exhibited an intermediate level of expression with a trend towards suppression by n-3 PUFA (HF versus HF-FO, *P* = 0.07). There was no effect of diet on FOXP3 mRNA expression (*P*>0.05).

**Table 2:** Colonic mucosal mRNA expression in TNBS-treated mice^1^.

Gene	HF	HF-FO	LF	*P*-value
RORγτ	2.3±0.3^a^	1.6±0.1^b^	1.3±0.2^b^	0.03
T-bet	3.6±1.9	2.1±0.2	1.1±0.4	0.07
Foxp3	2.8±0.7	2.1±0.4	1.2±0.5	0.18
IL-6	12.8±3.6^a^	2.2±0.7^b^	6.8±0.3^ab^	0.03
IL-1β	18.3±8.9	3.3±1.6	11.3±6.2	0.29
IL-17A	15.4±4.5^a^	5.5±2.1^b^	4.2±2.4^b^	0.05
IL-17F	4.4±1.0^a^	1.5±0.4^b^	2.8±0.4^ab^	0.01
IL-21	3.2±0.5^a^	0.8±0.2^b^	3.4±0.7^a^	0.03
IL-23	9.5±2.5^a^	5.4±0.8^b^	8.9±0.6^a^	0.04
IL-27	6.5±1.5	5.0±0.7	5.2±0.8	0.60
IFNγ	6.5±2.4^a^	1.3±0.2^b^	5.9±1.4^a^	0.05
IL-10	0.6±0.3^a^	1.8±0.4^b^	0.3±0.07^a^	0.03
TGF-β1	3.5±0.6	3.0±0.8	3.7±0.9	0.54

1 Values are means ± SEM (n = 6/TNBS-treated mice/dietary group). Data were analyzed by two-way ANOVA (main effects: diet and treatment). For all genes, only the effect of diet is shown (*P*≤0.05). Values not sharing a lower case letter differ. Data were normalized to ribosomal 18S.

Expression of colonic lamina propria cell-derived cytokines known to govern Th17 cell differentiation/polarization, proliferation and maintenance of a Th17 cell phenotype were also examined (**Table 2**). As expected, exposure to TNBS upregulated mucosal inflammatory gene expression relative to vehicle controls (*P*>0.05) in all dietary groups. Within TNBS-treated mice, the HF-FO diet reduced mucosal mRNA expression of Th17 cytokines, IL-17A and IL-17F, by 64% (*P* = 0.05) and 66% (*P* = 0.01), respectively, compared to HF. Similarly, HF-FO reduced IFNγ mRNA expression by 80% (*P* = 0.05) compared to HF. Additionally, dietary n-3 PUFA reduced IL-6 and IL-21 mRNA expression by 83% (*P* = 0.03) and 75% (*P* = 0.03), respectively, compared to HF. This is noteworthy because in combination with TGF-β, which was unaffected by diet (*P* = 0.54), IL-6 or IL-21 can drive Th17 cell differentiation [56]. Similarly, mucosal mRNA expression of IL-23, which contributes to Th17 cell expansion, stabilization and/or conditioning of a fully inflammatory cell phenotype [57], was reduced by 43% (*P* = 0.04) in HF-FO-fed mice relative to HF whereas mRNA expression of IL-1β and IL-27 were unaffected by diet (*P*>0.05). Conversely, consistent with the anti-inflammatory nature of n-3 PUFA, mucosal mRNA expression of IL-10 was elevated 67% (*P* = 0.03) in the HF-FO group versus HF. Collectively, these data indicate that during coincident obesity and TNBS-induced colitis, the inflammatory phenotype of the colonic mucosal cytokine microenvironment is suppressed by n-3 PUFA.

### n-3 PUFA Suppress Systemic Inflammatory Th17 and Th1 Cells Following TNBS Exposure

**Figure 4:**
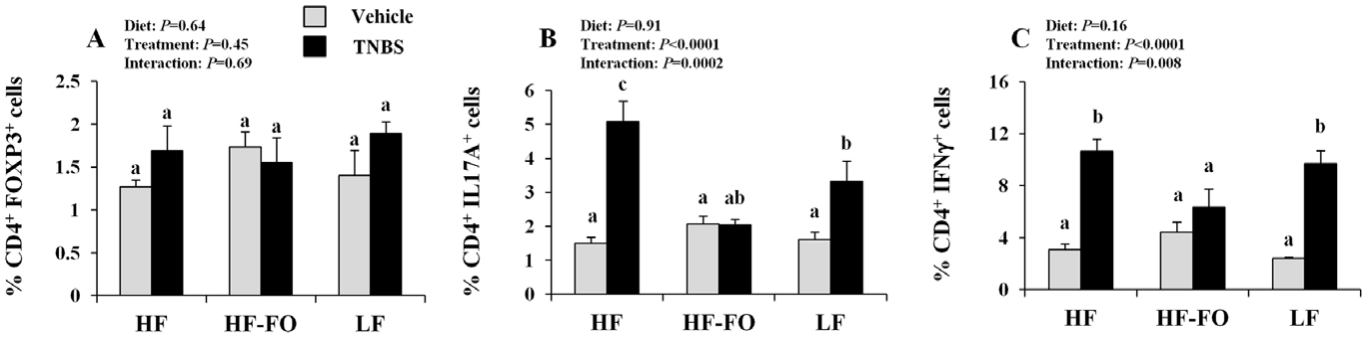
Effect of diet and colitis on splenic T cell subsets. A) Tregs (CD4^+^ FOXP3^+^), B) Th17 (CD4^+^ IL17A^+^), and C) Th1 (CD4^+^ IFNγ^+^) cell populations (n = 3–6 vehicle controls and n = 6−12 TNBS treated mice/dietary group). Bars represent mean values ± SEM. Bars not sharing a common letter are significantly different (P≤0.05).

Critical T cell subsets were monitored in the spleen, the central systemic lymphoid organ of the body that is responsible for propagating inflammatory immune responses following colitis induction, and representative dot plots for each T cell subset are shown in **Figure S2**. The percentage of splenic Tregs (CD4^+^ FOXP3^+^, **Figure 4A**) did not differ between dietary groups (*P* = 0.64) nor did this subset of cells exhibit a colitis-associated change in frequency (*P* = 0.45). Conversely, the percentage of splenic Th17 cells (CD4^+^ IL-17A^+^, **Figure 4B**) was elevated within both the HF and LF diet groups following the induction of colitis (within dietary groups vehicle control versus TNBS, *P*<0.05), which was not apparent in the HF-FO group (vehicle control versus TNBS-treated, *P* = 0.66). Moreover, the magnitude of the TNBS-associated induction in splenic Th17 cells was higher in the HF group relative to the LF, indicating that obesity promoted a colitis-associated Th17 cell response, as reported previously [36]. Within TNBS-treated mice, the HF-FO group exhibited a reduced percentage of Th17 cells relative to HF but did not differ from LF. Similarly, the percentage of inflammatory Th1 cells (CD4^+^ IFNγ^+^, **Figure 4C**) exhibited a TNBS-associated increase compared to vehicle controls in the HF and LF groups; however, this was prevented in the HF-FO group. Although the magnitude of the colitis-associated change in Th1 cells did not differ between the HF and LF groups, comparatively, HF-FO reduced the percentage of Th1 cells (*P*<0.05). Overall, these data indicate that n-3 PUFA suppress the systemic induction of two critical inflammatory T cell subsets that are strongly implicated in the pathogenesis of IBD [20].

**Figure 5:**
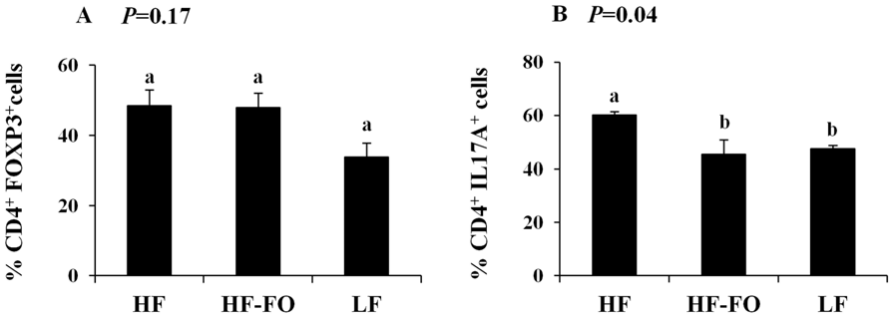
Effect of diet on splenic CD4^+^ T cell polarization. Splenic CD4^+^ cells were purified by positive selection and cultured for 3 d under A) Th17 or B) Treg polarizing conditions (see Materials and Methods, n = 3−4 TNBS treated mice/dietary group). Bars represent mean values ± SEM. Bars not sharing a common letter are significantly different (P≤0.05).

One potential mechanism underlying the observation of reduced splenic Th17 cells in the HF-FO group is the ability of n-3 PUFA to suppress T cell differentiation into a Th17 cell phenotype. To address this mechanism, splenic CD4^+^ T cells were purified from TNBS-treated mice and cultured under Treg and Th17 cell polarizing conditions ex vivo (**Figures 5A & B**). Clonal expansion of Treg cells was unaffected by diet (*P* = 0.17). Conversely, differentiation of CD4^+^ T cells into Th17 cells was altered by diet (*P* = 0.04), with an increased percentage of Th17 cells detected in the HF group versus LF. Moreover, Th17 cell polarization was reduced in the HF-FO group as compared to HF (*P*<0.05).

## Discussion

We examined the effects of n-3 PUFA supplementation on obesity-associated colitis severity using the TNBS model of chemically induced colitis which is considered to be representative of CD [48]. Since the low-grade chronic inflammation associated with obesity further complicates many disease states, elucidation of mechanisms through which dietary n-3 PUFA impact the clinical outcome of concurrent diet-induced obesity and colitis has translational utility. In the TNBS colitis model, obesity increases disease severity and exhibits a bias towards the activation of inflammatory Th17 cells [36], a T cell subset that plays a pathogenic role in IBD [18], [57]. Moreover, obesity worsens the clinical outcome of CD wherein adipose-derived inflammatory mediators perpetuate inflammatory responses [26], [32], [33], promoting a more aggressive clinical course [27], [31], [34], [35]. Focusing on three major tissue sites, namely the visceral adipose tissue (local site of obesity-associated inflammation and a major endocrine organ that is closely associated with the inflamed colon during colitis), colon (target tissue and site of local inflammation in colitis) and spleen (indicative of the systemic immunological and inflammatory phenotype), we were able to document changes in both the local and systemic inflammatory milieu while tracking changes in key immune cell populations driving the obesity-associated inflammatory response to colitis.

The HF-FO diet significantly impacted the obese phenotype in a manner consistent with reduced inflammation and improved metabolic outcomes. The blood hormone profile was improved as dietary n-3 PUFA reduced circulating levels of the inflammatory hormones leptin and resistin (**Figure 1C & D**), while increasing the levels of the anti-inflammatory hormone adiponectin relative to the HF group (**Figure 1E**). These results confirm previous findings in n-3 PUFA supplemented rodent diet-induced obesity models [**5]**, [6], [12] and extend the n-3 PUFA modifiable blood hormone profile to include resistin, an inflammatory adipokine that is increased in both the serum and adipose tissue in obese humans [49], [58].

Obesity-induced adipose tissue inflammation is a unique process characterized by a broad panel of cytokines [37], [59]. The obesity-related adipose gene expression pattern was dramatically altered by the HF-FO diet (**Table 1**), whereby expression of MCP-1, IL-6 and IFNγ mRNA levels were reduced and IL-10 mRNA expression was elevated, compared to HF. Moreover, mRNA levels of cytokines related to the inflammatory Th17 cell population, IL-17F and IL-21, were also reduced by n-3 PUFA. Lastly, mRNA expression of adipose derived T cell-associated transcription factors specific for particular inflammatory T cell subsets Tbet (Th1 cells) and RORγτ (Th17 cells) were significantly reduced or exhibited a non-significant trend towards reduction, respectively, by n-3 PUFA.

A hallmark of obesity is the increased infiltration of macrophages into the inflamed adipose tissue [51], [52], [53]. Interestingly, n-3 PUFA reduced the percentage of adipose tissue macrophages (F4/80^+^ CD11b^+^) relative to the HF diet (**Figure 2A**), consistent with the effect of n-3 PUFA on reducing MCP-1 gene expression (**Table 1**). Macrophages are broadly characterized as M1 or M2 by their polarization or activation state [60]. The M1 designation denotes classically activated macrophages (i.e., following stimulation with IFNγ and LPS), which exhibit an inflammatory phenotype (secrete high levels of TNFα, IL-6, IL-1β and MCP-1) and gene expression profile that differs from alternatively activated M2 macrophages which exhibit an anti-inflammatory phenotype and support tissue repair, remodeling and inflammation resolution via secretion of components of the extracellular matrix and IL-10 [44], [45], [53], [61]. In obesity, adipose tissue macrophages seemingly undergo a phenotypic switch from the M2 to the M1 phenotype [51], [52]. However, the precise mechanism underlying this observation is unknown and could be a result of newly recruited M1 macrophages to the adipose from the circulation, differentiation of resident M2 macrophages into the M1 phenotype, or both. Interestingly, our data indicate that the obesity-associated increase in adipose M1 macrophages can be prevented by the HF-FO diet (**Figure 2B**). There was no obesity-associated change in the percentage of adipose M2 macrophages (HF vs. LF); however, the percentage of M2 cells in the HF-FO group was significantly reduced relative to both HF and LF (*P* = 0.011, **Figure 2C**). Collectively, these results demonstrate that supplementation of n-3 PUFA to a high fat diet reduces total adipose macrophage infiltration, thereby reducing the percentage of both the M1 and M2 subsets. The reduction in M1 macrophage number, a predominant cellular source of inflammatory adipokines [44], [45], [51], [61], may explain the reduced mRNA levels of IL-6 and MCP-1 in the HF-FO group. Overall, the HF-FO diet dramatically ameliorated the adipose tissue inflammatory phenotype; including reducing (i) circulating levels of adipocyte derived inflammatory hormones (leptin and resistin), (ii) adipose inflammatory cytokine mRNA expression and (iii) total macrophage infiltration and the percentage of inflammatory M1 cells.

Obesity increases the severity of mucosal damage associated with TNBS-induced colitis [36], and we confirmed this finding by observing that colon injury and inflammation scores were higher in the HF group compared to LF. A key novel finding of this study was the ability n-3 PUFA supplementation to ameliorate the obesity-associated increase in colon architectural damage (*P* = 0.05, **Figure 3E**). This beneficial effect of n-3 PUFA has been observed previously in the chronic DSS colitis model [25] and is consistent with the ability of dietary n-3 PUFA to promote long-term resolution of colon inflammation and mucosal repair [42], [62]. Furthermore, we monitored the inflammatory milieu based on changes in gene expression within the colonic mucosal microenvironment with a particular focus on Th17 cell-related cytokine targets, as the combination of obesity and TNBS-induced colitis is associated with a bias towards Th17 cells [36]. In TNBS-treated mice, the obesity-associated increase in colonic mRNA levels of Th17-associated cytokines was suppressed by n-3 PUFA (**Table 2**). Thus, colonic mucosal mRNA expression of IL-17A, IL-17F, IL-6, IL-21, IL-23 and INFγ was reduced in the HF-FO group compared to HF. Consistent with the reduced activation and presence of inflammatory T cell subsets within the colon following colitis induction, mRNA expression of the Th17 cell specific transcription factor RORγτ was significantly reduced by n-3 PUFA, whereas Tbet mRNA levels showed a non-significant trend towards suppressed colonic expression. Therefore, on an mRNA level, signature cytokines of inflammatory T cell subsets involved in the pathogenesis of colitis [18], [20] are significantly reduced by dietary n-3 PUFA. Lastly, n-3 PUFA upregulated mRNA expression of the anti-inflammatory cytokine, IL-10, which suppresses colonic inflammation [63] and is capable of suppressing Th17 cells [64], [65]. These findings confirm previous reports that n-3 PUFA suppress multiple aspects of Th17 cell mucosal biology during chronic colitis [25] and collectively demonstrate that n-3 PUFA modulate the colonic cytokine microenvironment in a manner that is less compatible with the activation, polarization, proliferation, maintenance and function of pathogenic inflammatory Th17 cells [18], [25], [56].

We demonstrate for the first time that the combined obesity and colitis-associated increase in the percentage of inflammatory splenic Th17 and Th1 cells is reduced by n-3 PUFA (**Figure 4B&C**). These data provide direct evidence that dietary n-3 PUFA can prevent the obesity-associated Th17 cell bias. When splenic CD4^+^ T cells were cultured under Th17 cell polarizing conditions (**Figure 5A**), n-3 PUFA rendered the cells more refractive towards polarization signals. Further studies are required to determine the mechanism(s) by which n-3 PUFA suppress Th17 polarization.

In summary, we combined established models of high fat diet induced obesity and colitis to demonstrate the ability of dietary long chain n-3 PUFA to ameliorate disease progression, in part, by reducing inflammatory cytokine gene expression and immune cell populations both locally and systemically. The obesity-associated bias towards inflammatory Th17 cells was prevented by n-3 PUFA supplementation. Both the adipose and colonic mucosal gene expression profiles were reconfigured in a manner consistent with reduced inflammatory capacity and the suppression of Th17 cell activation and function. Further studies are required to determine the utility of dietary n-3 PUFA as an anti-IL-17 and/or anti-IFNγ therapy for treating chronic inflammatory diseases.

## Supporting Information

Figure S1 Representative dot plots from high fat (HF) TNBS-treated mouse visceral adipose tissue stromal vascular cells. A) Total macrophages (F4/80^+^ CD11b^+^), B) M1 macrophages (F4/80^+^ CD11c^+^), and C) M2 macrophages (F4/80^+^ CD206^+^).
(TIF)

Figure S2 Representative dot plots of splenic T cell subsets isolated from high fat (HF) TNBS-treated mice. T cell subsets were identified within a mononuclear cells suspension by a combination of surface (CD4^+^) and intracellular staining. A) Tregs (CD4^+^ FOXP3^+^), B) Th17 cells (CD4^+^ IL17A^+^) C) Th1 cells (CD4^+^ IFNγ^+^). CD4^+^ T cells were purified by positive selection (Miltenyi Biotec) and cultured for 72 h under Treg or Th17 cell polarizing conditions (see Materials and Methods). Representative dot plots of polarized D) Tregs (CD4^+^ FOXP3^+^) and E) Th17 cells (CD4^+^ IL17A^+^) are shown.
(TIF)

Table S1 Semi-purified diet composition**.** All diet constituents were purchased from Bio Serv (Bio Serv, Frenchtown, NJ), except lard (ConAgra Foods, Omaha, NE) corn oil (Dyets, Madison, WI) and fish oil (Omega Protein Inc, Reedville, VA).
(PDF)

Table S2 Colonic mucosal mRNA expression in vehicle control treated mice. Values are means ± SEM (n = 4−6/vehicle control mice/dietary group). Data were normalized to ribosomal 18S and analyzed by ANOVA. For all genes, the effect of diet is shown (significance *P*≤0.05).
(PDF)
