# Supplementary figures and images for: Dietary n-3 Polyunsaturated Fatty Acids (PUFA) Decrease Obesity-Associated Th17 Cell-Mediated Inflammation during Colitis
Source: PLoS One. 2012 Nov 16;7(11):e49739. doi: 10.1371/journal.pone.0049739 (PMC3500317; doi:10.1371/journal.pone.0049739)

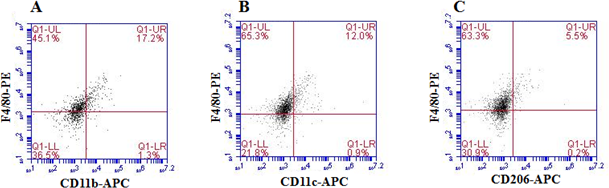

Supplement: Figure S1 — Representative dot plots from high fat (HF) TNBS-treated mouse visceral adipose tissue stromal vascular cells. A) Total macrophages (F4/80+ CD11b+), B) M1 macrophages (F4/80+ CD11c+), and C) M2 macrophages (F4/80+ CD206+). (TIF) [file pone.0049739.s001.tif]

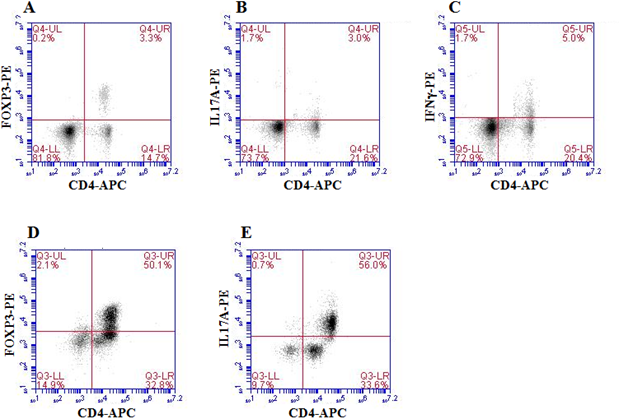

Supplement: Figure S2 — Representative dot plots of splenic T cell subsets isolated from high fat (HF) TNBS-treated mice. T cell subsets were identified within a mononuclear cells suspension by a combination of surface (CD4+) and intracellular staining. A) Tregs (CD4+ FOXP3+), B) Th17 cells (CD4+ IL17A+) C) Th1 cells (CD4+ IFNγ+). CD4+ T cells were purified by positive selection (Miltenyi Biotec) and cultured for 72 h under Treg or Th17 cell polarizing conditions (see Materials and Methods). Representative dot plots of polarized D) Tregs (CD4+ FOXP3+) and E) Th17 cells (CD4+ IL17A+) are shown. (TIF) [file pone.0049739.s002.tif]
