# Supplementary material for: Dietary n-3 Polyunsaturated Fatty Acids (PUFA) Decrease Obesity-Associated Th17 Cell-Mediated Inflammation during Colitis
Source: PLoS One. 2012 Nov 16;7(11):e49739. doi: 10.1371/journal.pone.0049739 (PMC3500317; doi:10.1371/journal.pone.0049739)
Supplement: Table S1 — Semi-purified diet composition. All diet constituents were purchased from Bio Serv (Bio Serv, Frenchtown, NJ), except lard (ConAgra Foods, Omaha, NE) corn oil (Dyets, Madison, WI) and fish oil (Omega Protein Inc, Reedville, VA). (PDF) [file pone.0049739.s003.pdf]

Table S1. Semi-purified diet composition[1]

Diet Constituent (g/kg diet)	HF	HF-FO	LF
Casein	200	200	200
Methionine	3	3	3
Sucrose	338	338	338
Corn Starch	0	0	306
Cellulose	60	60	60
AIN-76 Mineral Mix	35	35	35
Vitamin Mix	13	13	13
Choline Chloride	2	2	2
Lard	230	230	19
Corn Oil	119	33	24
Fish Oil	0	86	0

[1]All diet constituents were purchased from Bio Serv (Bio Serv, Frenchtown, NJ), except lard (ConAgra Foods, Omaha, NE) corn oil (Dyets, Madison, WI) and fish oil (Omega Protein Inc, Reedville, VA).
