# Supplementary material for: Dietary n-3 Polyunsaturated Fatty Acids (PUFA) Decrease Obesity-Associated Th17 Cell-Mediated Inflammation during Colitis
Source: PLoS One. 2012 Nov 16;7(11):e49739. doi: 10.1371/journal.pone.0049739 (PMC3500317; doi:10.1371/journal.pone.0049739)
Supplement: Table S2 — Colonic mucosal mRNA expression in vehicle control treated mice. Values are means ± SEM (n = 4−6/vehicle control mice/dietary group). Data were normalized to ribosomal 18S and analyzed by ANOVA. For all genes, the effect of diet is shown (significance P≤0.05). (PDF) [file pone.0049739.s004.pdf]

Table S2. Colonic mucosal mRNA expression in vehicle control treated mice[1]

**Gene**	**HF**	**HF-FO**	**LF**	***P*-value**
RORγτ	1.9 ± 0.3	1.4 ± 0.1	1.1 ± 0.2	0.16
T-bet	2.5 ± 0.2	2.7 ± 0.4	2.4 ± 0.9	0.62
Foxp3	0.2 ± 0.02	0.5 ± 0.2	0.5 ± 0.3	0.39
IL-6	0.6 ± 0.2	0.6± 0.2	0.6 ± 0.01	0.25
IL-1β	3.0 ± 1.3	3.4 ± 1.7	3.6 ± 1.7	0.96
IL-17F	0.9 ± 0.2	0.5 ± 0.2	0.5 ± 0.3	0.21
IL-21	0.42 ± 0.2	0.2 ± 0.1	0.2 ± 0.09	0.44
IL-23	1.0 ± 0.1	1.6 ± 0.6	0.7 ± 0.07	0.29
IL-27	4.2 ± 2.3	5.0 ± 1.9	8.6 ± 1.4	0.14
IFNγ	0.3 ± 0.1	0.4 ± 0.1	0.8 ± 0.2	0.37
IL-10	0.9 ± 0.2	1.2 ± 0.4	0.2 ± 0.03	0.33
TGF-β1	3.3 ± 0.5	4.8 ± 0.9	3.9 ± 0.9	0.15

[1]Values are means ± SEM (n=4-6/vehicle control mice/dietary group). Data were normalized to ribosomal 18S and analyzed by ANOVA. For all genes, the effect of diet is shown ($P \leq 0.05$).
